# How to Conduct a Dental Practice in an Agricultural District

**Published:** 1897-10

**Authors:** Burt Abell

**Affiliations:** Albion, Mich.


### How to Conduct a Dental Practice in an Agricultural District.

BY DR. BURT ABELL, D.D.S., OF ALBION, MICH.

When Dr. Diack's gentle request came to me with, "You've *got* to take this subject, I can't let you off," I was *very* happy for of course I know all about this subject and was anxious to tell you all about it. I am peculiarly fitted for the task also, having been raised on a farm and practiced for three whole long years in the north woods in a country town consisting of a saw-mill, post-office and corner grocery with a few scattered wigwams of the festive red man who frequently borrowed my last ten cents to buy corn. I've learned since that it was in the liquid form he obtained it. Perhaps it's rather malapropos to mention him as *he* did the practicing on *me*. He practiced on my credulity. But as Samantha Allen says, "To resume and continue on." There next came to me the thought of how chagrined some of you would feel because you had practiced all these years and had never before asked me to tell you how it should be done. I was afraid when the thought of so much misspent time fully dawned upon you that you would rise as one man and slay Dr. Diack for ever having caused you such a rude awakening. So I stated the case to some of my friends asking them to give me pointers as to what I should say. Dr. W. G. Stevenson, of Morenci, Dr. Wm. R. Calhoun, of Milan, Dr. Wm. M. Welch, of Homer and Dr. H. C. Rockwell, of Benton Harbor, all took pity on *you* and lent their aid to straighten things out, *provided* I wouldn't give them away as to which thing each had said. I readily agreed to this as it gave me the opportunity of throwing on their shoulders the responsibility for anything displeasing to you should I be taken to task for it. There's *nothing* I admire so much as *gratitude*.

If you please, we will consider this question in the first person to be sure we tread on nobody's toes.

When we left college and began our practice on an unsuspecting public, we were just jam full of high ideas of how everything ought to be done and were firmly resolved never to vary from them. But one day there came into our office a fellow from away off, fifteen miles in the country, having trouble with a pulpless tooth. He wants it saved and is ready to have anything done that won't take more than an hour or two, and here we want to see him from three to seven times to get it ready to fill, never having heard of Wild's method (or Dr. White's sulphuric acid treatment) of immediately settling such cases. He's an exception to the average countrymen if he considers that tooth worth traveling from 45 to 100 miles to save, to say nothing of the extra expense of treatment, so out comes a good tooth and down like a nine-pin drops one of our high ideas of saving everything, and when he is followed by a mother who says, "Take his teeth out, they are only baby teeth, anyway," and she by a patient with a set of teeth requiring three hours to scale according to our idea and he grumbles when we charge him seventy-five cents. The whole set of ideas are in danger of coming down with the rushing fall of necessity. We have to learn in a country practice to do the best we can under the circumstances, no matter what we'd like to do.

We as country dentists do not have that delightful privilege of sorting out the best and sending the rest to some one else that our city brothers have. All must be grist that comes to our mill. Yet we can *begin* right by securing the patronage of the best families and as large a circle of acquaintances as possible. I might quote right here, "If you would have friends, show yourself friendly." Dental services in the city are no ground for social position, but our country patient expects us to speak when we meet him and thinks us a snob if we fail to do so. To enlarge our circle of acquaintances we should attend church regularly, and if Christian men, be active in Christian work. I believe every dentist ought to be a Christian, and then our church, social and professional work will be done for the Master's sake, and we have his promise that if we "seek first the kingdom, all these things shall be added." At the least, we should be morally and personally the purest kind of men. In our country practice we come in contact with so much of uncouth humanity. The temptation is strong to drift into slackness in our personal habits. I remember Dr. Taft telling us in college about an old negress, who, after visiting a dentist, said, "Why, he washed his hands just for me," which so pleased the mistress that the whole family became his patients. At least, one has the satisfaction of knowing he is clean whether or not his patients appreciate it. We may be sure there are at least *some* who will.

It's just as incumbent upon the country dentist as the city practitioner to be conscientious in all he does. As I said before, circumstances may prevent our doing as we might think was best, but we should satisfy our consciences at all times. If we break a tooth, tell the patient so. If we know we can't do a piece of work justice, leave it alone. We'll lose nothing by being true to the best there is in us.

Country people don't care to hear us discourse about our affairs, but fully appreciate our lively interest in how Johnny is getting along since his accident, or the fact that we have seen something in the paper pertaining to them. We should make them feel that our only interest in them is not for the dollars they pay us.

Another thing they will appreciate is that we suit our appointments to their convenience as much as possible. They should be given to understand they are expected at a certain time and that it will be reserved for them, but the hour should be one when they can best reach the office. I rather doubt whether a dentist could continue to work for a country patient against whom he had made a charge for time engaged and not filled. They have no idea of the value of time when reduced to hours and minutes, and they can't see what difference an hour or two makes, any way. We will find exceptions, of course, but only enough to prove the rule.

Farmers think they have to work pretty hard for their money and want the most they can get for a given amount. Consequently, a man who, with inferior work, can make a better showing will "get away" with the operative expert who stands on his professional dignity and charges a round fee for his services. It's a problem how to make them think we work cheaply and yet get *properly* paid for our services; a perfectly legitimate proceeding, provided we succeed, otherwise the veracity of our statements is called in question.

Competition is often as sharp in the country as in the city and with less good feeling, yet it is every man's privilege by careful work and courteous treatment to make his patrons think he is *the* dentist and not the *dentist*.

There are times of year when our farmer friends have less money than at others. Especially is this so in the fruit districts. This necessitates a credit system that is a constant annoyance to us dentists with small means, and a great many of us seem to have run up against this microbe of poverty and been pretty well infected. I wish those who have escaped would tell us what disinfectant they use.

Farmers are shoppers. They demand a foreknowledge of what their work will cost and it's best to estimate for them. Dr. Stevenson suggests a plan of a minimum price of say $20, a maximum of $30, leaving us a margin for errors in judgment, and we can often get $25 with satisfaction to all concerned. Oftentimes their idea of the cost of an operation would scarcely cover the cost of material.

We are not as busily engaged every hour in the day as our city brother is; the temptation is strong to be absent from the office at irregular hours. A good rule is to have *regular* office hours and *keep* them. We are often given to complaining because business is dull but it does no good. In Aurora Leigh I found this, "Let us be content in work, to do the thing we can and not presume to fret because it's little." We have more time to talk politics and swap fish stories and it does'nt hurt us if we allow our patients to think they have the best of the argument, and it's a mean thing to tell a bigger fish story than anybody else, anyway. But if we don't look out we shall have our office full of loungers a good share of the time and then when our village patients come in they immediately conclude to visit our competition where no such condition of affairs exists.

In country districts, people, as a rule, appreciate their teeth less than any other blessing with which an all-wise providence has endowed them. This may be due to the fact that substitutes are so easily obtained or lack of pride in personal appearance, or a knowledge of what may be done for the natural teeth. With this fact daily brought to our notice we must agree with Dr. T. W. Brophy when he says, "The most important place a dentist fills in the community in which he lives is that of teacher. I believe that 80 percent of the people, perhaps more, have no knowledge of the laws of hygiene as applied to the oral cavity; and believing this as I do, I desire to see dentists and physicians active in informing people in regard to this important subject.

No one will question the breadth of the dentist's sphere, if I am right. If 80 percent of the American people require instruction in oral hygiene and in regard to the diseases of the teeth, 48,000,000 are uninformed." A glance into the oral cavity of some of our patients is enough to convince us. What can we expect when they tell us, "why, yes, I clean my teeth, always once a week, sometimes twice."

Some of the most erroneous ideas are current of even the anatomy of oral structures; double teeth all around, requests to fill spaces between teeth with gold, and insert teeth without plates when not a root is left, and so on, ad infinitum.

Who is to teach the mothers the care of the children's teeth if not the dentist? Who is to instruct the people as to the value of living tooth structure if we neglect this most important part of our duties? Every patient should be told the exact condition of his teeth even though he is in the chair for no more than an extraction. We should fight to save teeth that can be made of service, call his attention to bridge-work instead of plates where bridges are better. Send patients a notice that six months have passed since their teeth have been examined as shown by our records and in every way look after their interests.

How shall we country dentists teach the people what is best if we ourselves neglect our own education? Who of us will dare to trust our college course to tide us over the hard places? A college course is a most excellent foundation, but, as was said at Kalamazoo last April, these conventions are now our colleges, our text-books, the papers and discussions, and our reference library, our journals.

Brethren, let us be abreast of the times and not shut ourselves up in our little corners, but come out into the full sunshine of truth and knowledge and with a fraternal spirit of mutual helpfulness aid in the advancement of the profession and the betterment of humanity, confident that the good of the whole means the welfare of each.

---

A PERSON once convicted of felony can never practice medicine in Ohio or New York.

